# Hyperuricemia in Children and Adolescents with Autism Spectrum Disorder Treated with Risperidone: The Risk Factors for Metabolic Adverse Effects

**DOI:** 10.3389/fphar.2016.00527

**Published:** 2017-01-05

**Authors:** Natchaya Vanwong, Pornpen Srisawasdi, Nattawat Ngamsamut, Nopphadol Nuntamool, Apichaya Puangpetch, Bhunnada Chamkrachangpada, Yaowaluck Hongkaew, Penkhae Limsila, Wiranpat Kittitharaphan, Chonlaphat Sukasem

**Affiliations:** ^1^Division of Pharmacogenomics and Personalized Medicine, Department of Pathology, Faculty of Medicine Ramathibodi Hospital, Mahidol UniversityBangkok, Thailand; ^2^Laboratory for Pharmacogenomics, Somdech Phra Debaratana Medical Center, Ramathibodi HospitalBangkok, Thailand; ^3^Division of Clinical Chemistry, Department of Pathology, Faculty of Medicine, Ramathibodi Hospital, Mahidol UniversityBangkok, Thailand; ^4^Yuwaprasart Waithayopathum Child and Adolescent Psychiatric Hospital, Department of Mental Health Services, Ministry of Public HealthSamut Prakarn, Thailand; ^5^Molecular Medicine, Faculty of Science, Mahidol UniversityBangkok, Thailand

**Keywords:** serum uric acid levels, risperidone, autism spectrum disorder, hyperuricemia, metabolic adverse effects

## Abstract

**Background:** Atypical antipsychotics have been found to be associated with hyperuricemia. Risperidone, one of the atypical antipsychotics, might be related to the hyperuricemia among autism spectrum disorder (ASD) patients. The aims of this study were to determine the prevalence of hyperuricemia in ASD patients treated with risperidone and to determine associations between serum uric acid levels and risperidone dosage, treatment duration, and metabolic parameters.

**Methods:** 127 children and adolescents with ASD treated with risperidone and 76 age-matched risperidone-naïve patients with ASD were recruited. The clinical data and laboratory data were analyzed. Hyperuricemia was defined as serum uric acid >5.5 mg/dl.

**Results:** Hyperuricemia was present in 44.70% of risperidone-naïve patients with ASD and 57.50% of ASD patients treated with risperidone. The fasting uric acid levels were significantly higher in the risperidone group than in the risperidone-naïve group (5.70 vs. 5.35 mg/dl, *P* = 0.01). The increased uric acid concentrations were significantly associated with adolescent patients treated with risperidone. The higher dose of risperidone and/or the longer treatment time were associated with the increased uric acid levels. Uric acid levels significantly rose with body mass index (BMI), waist circumference (WC), triglyceride (TG) levels, triglycerides to high-density lipoprotein cholesterol ratio (TG/HDL-C), insulin levels, homeostatic model assessment index (HOMA-IR), high-sensitivity CRP (hs-CRP) levels, and leptin levels. Conversely, the levels of HDL-C and adiponectin were negatively correlated with uric acid levels. In multiple regression analysis, there were age, BMI, TG/HDL-C ratio, and adiponectin levels remained significantly associated with uric acid levels.

**Conclusion:** Hyperuricemia may play a role in metabolic adverse effect in children and adolescents with ASDs receiving the high dose and/or the long-term treatment with risperidone.

## Introduction

Uric acid is the final oxidation product of the purine degradation in humans. Uric acid at normal plasma levels acts as a free-radical scavenger, which contributes to the neuroprotective effect. Studies, however, have demonstrated that an excessive amount of uric acid is correlated with the risk of cardiovascular disease (Hongo et al., 2010; Trepiccione and Perna, 2015). Hyperuricemia has been associated with abdominal obesity (Godin et al., 2015), inflammation (Baldwin et al., 2011), type 2 diabetes (Dehghan et al., 2008), dyslipidemia (Peng et al., 2015), and metabolic syndrome (Sagodi et al., 2015). An excessive amount of uric acid is considered to be a mediator of proinflammatory endocrine imbalance in adipose tissue, contributing to the development of dyslipidemia and low-grade inflammation (Baldwin et al., 2011). Patients with autism spectrum disorder (ASD) may have increased levels of uric acid (Page and Coleman, 2000; Jinnah et al., 2013; Lindberg et al., 2015). Mitochondrial and purinergic dysfunction have been associated with mental illnesses such as bipolar disorder, ASD, and schizophrenia (Lindberg et al., 2015). A previous study found increased levels of serum uric acid in adolescent patients with bipolar mania when treated with an atypical antipsychotic, olanzapine (Tohen et al., 2007). and this may enhance increased cardiovascular risk when using antipsychotics as treatment.

Atypical antipsychotic agents are widely used psychopharmacological interventions for autism spectrum disorder (ASD). Among the atypical antipsychotic agents, risperidone has demonstrated considerable benefits in reducing several behavioral symptoms associated with ASD. Previous studies reported that treatment with olanzapine was associated with the increase in uric acid concentrations (Tohen et al., 2007, 2008; Kryzhanovskaya et al., 2009). There is, however, no report of the prevalence of hyperuricemia in ASD patients treated with risperidone and how risperidone affects the uric acid levels in ASD patients.

The purposes of this study were to determine the prevalence of hyperuricemia in ASD patients treated with risperidone and to determine the association between serum uric acid levels and risperidone dosage, treatment duration, and metabolic parameters.

## Materials and Methods

### Subjects and Study Design

Children and adolescent patients with ASD were recruited from the Yuwaprasart Waithayopathum Child Psychiatric Hospital, Samut Prakan, Thailand. One hundred and twenty seven patients with ASD treated with risperidone made up the case group. These patients were on risperidone more than 12 months prior to assessment. The control group was 76 risperidone-naïve patients with ASD, age-matched with patients in the case group. Regarding the risperidone-treated group, the recommended risperidone dosing for pediatric indications from the U.S. Food and Drug Administration (FDA) was adjusted according to weight; low dose, recommended dose, and high dose. The recommended dose of risperidone is 0.25–0.5 mg/day for patients with a body weight less than 20 kg and 0.5–1 mg/day for patients with body weight equal to or greater than 20 kg. Risperidone administration lower than the FDA recommendation was classified as the low dose, while risperidone dosage higher than FDA recommendation was classified as the high dose. Patients were classified into four groups based on the period of risperidone administration: Group 1 (0–24 months), Group 2 (>24 to 60 months), Group 3 (>60 to108 months), and Group 4 (>108 months). The nursing staff confirmed patient compliance. All the adolescent patients of 3–20 years were diagnosed as ASD according to the Diagnostic and Statistical Manual of Mental Disorders, fourth edition. Risperidone-treated patients or risperidone-naïve patients receiving concomitant drugs that could potentially affect the level of uric acid or other metabolic parameters were excluded from the study. Parents of all the patients involved in the study gave informed written consent to participate. The Ramathibodi Ethics Committee, Bangkok, Thailand, approved this study. Anthropometric measurements and biomedical parameters were recorded and analyzed in the risperidone-treated group. Body mass index (BMI) was calculated as body weight in kilograms divided by the height in meter squared (kg/m^2^).

### Serum Uric Acid and Biochemical Parameters Measurement

Blood samples for the chemistry panel were collected in the morning after overnight fasting. The samples were immediately transferred to the central laboratory where they were analyzed. Serum uric acid, glucose, and lipid profiles were determined by the colorimetric method using automated equipment (FUJI DRI-CHEM 4000i system, Germany). Hyperuricemia was defined as the level of uric acid concentration in the blood >5.5 mg/dL (Feig and Johnson, 2003). Insulin levels were measured by enzyme-labeled chemiluminescent immunoassay in automated equipment (IMMULITE^®^2000 System-SIEMENS, Germany). The homeostatic model assessment index (HOMA-IR) was calculated using the formula: HOMA-IR = [fasting glucose (mg/dl) × insulin (μIU/ml)]/405. The sandwich-ELISA assay in automated equipment (BEP^®^ III System – SIEMENS, Germany) measured leptin and adiponectin. Nephelometric technology using automated equipment (Siemens BN ProSpec^®^ System, SIEMENS, Germany) measured High sensitive C-reactive protein (hs-CRP).

### Statistical Analysis

Data were analyzed using SPSS version 16.0 (SPSS Inc., Chicago, IL, USA). Descriptive statistics were used to describe the clinical characteristics of the subjects. Discrete variables are expressed as counts (percentage) and continuous variables as median with the interquartile range (Q_1_–Q_3_). The nonparametric Mann–Whitney *U* test compared differences in median values (interquartile range) between the two groups, and Kruskal–Wallis test was used to make comparisons between three groups. The Chi-squared test was used for comparisons between two categorical variables. Accordingly, for nonparametric data, the Spearman rank correlation test measured relationships between two continuous random variables. Stepwise multiple linear regression analysis was used to adjust variables. Statistical significance was set at *P* < 0.05.

## Results

**FIGURE 1:**
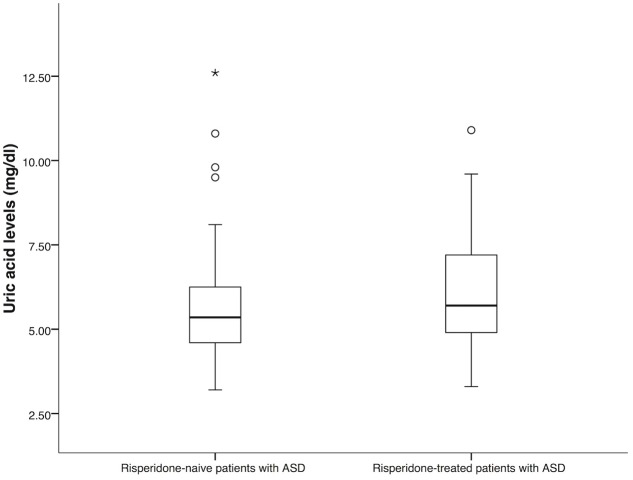
**Comparison of uric acid levels between risperidone-naïve patients with ASD (*n* = 76) and risperidone-treated patients with ASD (*n* = 127).** Statistical significance was calculated by Mann–Whitney *U* Test; ^∗^ *P*-value < 0.05.

The demographic and clinical characteristics of the patients were summarized in **Table 1**. There was no significant difference between the group of risperidone-naïve patients and risperidone-treated patients in age and gender (*P* = 0.15 and *P* = 0.09). In both groups, most of the patients were male. For the group of risperidone-naïve patients, the serum uric acid levels were 5.35 mg/dl (IQR: 4.60–6.28) and 5.70 mg/dl (IQR: 4.90–7.20) for the patients treated with risperidone. There were significant differences between the groups in uric acid levels (*P* = 0.01, **Figure 1**). Hyperuricemia was found in 34 (44.70%) patients from the risperidone-naïve group and 73 (57.50%) subjects from the risperidone group (**Table 1**).

**Table 1:** Demographic and clinical characteristics of subjects.

*Characteristics*	*Naïve-Risperidone, n = 76*	*Risperidone, n = 127*	*P-value*
Age-group			0.15^b^
Children, *n* (%)	42 (55.3%)	57 (44.9%)	
Adolescents, *n* (%)	34 (44.7%)	70 (55.1%)	
Gender, *n* (%)			
Male	61 (80.3%)	113 (89.0%)	0.09^b^
Female	15 (19.7%)	14 (11.0%)	
Uric acid levels (mg/dl)	5.35 (4.60–6.28)	5.70 (4.90–7.20)	0.01^∗a^
Hyperuricemia			0.08^b^
(Uric acid > 5.5 mg/dl), *n* (%)	34 (44.70%)	73 (57.50%)	
(Uric acid ≤ 5.5 mg/dl), *n* (%)	42 (55.30%)	54 (42.50%)	
Risperidone dose (mg/day)	–	1.00 (0.50–1.50)	–
Dose of risperidone treatment, *n* (%)			
Low dose	–	13 (10.24%)	–
Recommended dose	–	68 (53.54%)	
High dose	–	46 (36.22%)	
Duration of treatment (months)	–	61.27 (39.83–87.97)	–
Duration of treatment, *n* (%)			
0–24 months		6 (4.72%)	
>24–60 months	–	50 (39.37%)	–
>60–108 months	–	52 (40.95%)	
>108 months	–	19 (14.96%)	
Medication regimen			
Single risperidone	–	48 (37.80%)	–
Concomitant therapy	–	79 (62.20%)	


*^a^Statistical significance was calculated by Mann–Whitney *U* Test.*

*^b^Statistical significance was calculated by Chi-squared test.*

*^∗^*P*-value < 0.05.*

**FIGURE 2:**
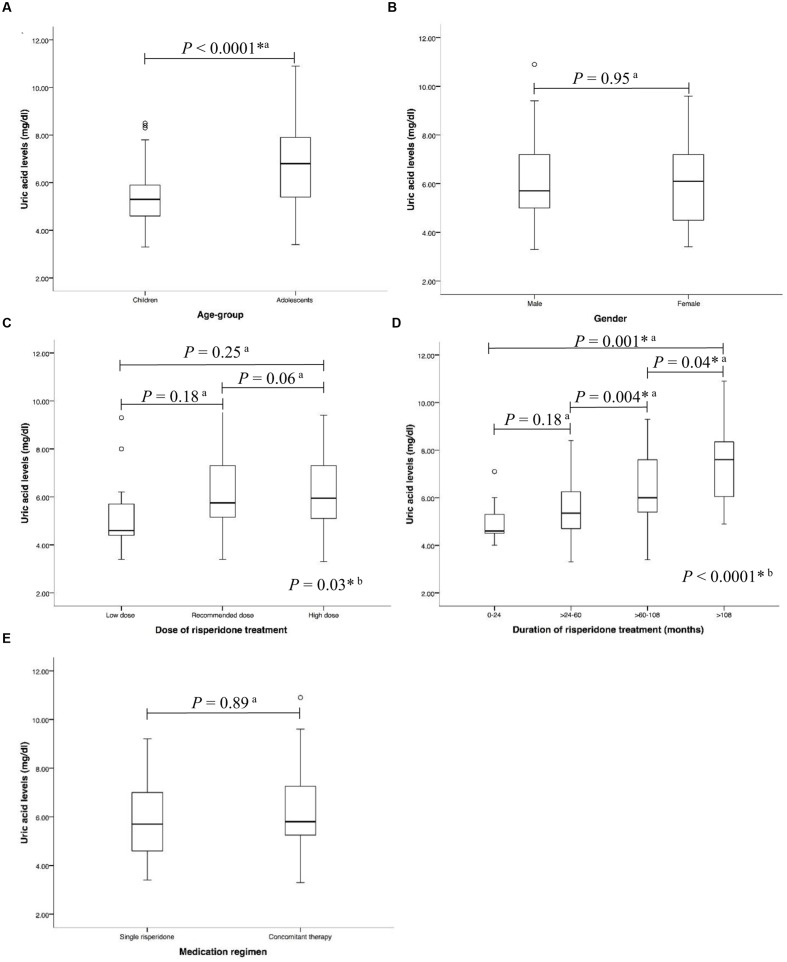
**Associations between uric acid levels and** **(A)** age-group **(B)** gender **(C)** dose of risperidone treatment **(D)** duration of risperidone treatment, and **(E)** medication regimen in risperidone-treated patients with ASD (*n* = 127) a, Statistical significance was calculated by Mann–Whitney *U* Test; b, Statistical significance was calculated by Kruskal–Wallis test ^∗^*P*-value < 0.05.

In the risperidone group, the median risperidone dose of the subjects was 1 mg/ day (IQR: 0.50–1.50). Sixty-eight (53.54%) patients were treated with risperidone at the FDA-recommended dose. Forty-six (36.22%) of them have been provided with higher than the FDA-recommended doses, while thirteen (10.24%) patients received lower than the FDA-recommended dose. The median duration of risperidone treatment was 61.27 months (IQR: 39.83–87.97). Most patients received risperidone for >60–108 months (>5–9 years) (**Table 1**). Forty-eight (37.80%) received only risperidone, the remaining patients received risperidone-containing concomitant therapy (62.20%). Median uric acid levels were significantly higher in the adolescents than in those children (6.80 mg/dl (IQR: 5.40–7.90) vs. 5.30 mg/dl (IQR: 4.60–5.95), *P* < 0.0001), **Figure 2A**. The significant associations between serum uric acid concentrations and the dose of risperidone and the duration of risperidone treatment were found. The results demonstrated that there were statistically significant differences in uric acid levels between risperidone dose groups: low dose, recommended dose, and high dose (4.60 mg/dl (IQR: 4.20–5.95) vs. 5.75 mg/dl (IQR: 5.13–7.35) vs. 5.95mg/dl (IQR: 5.08–7.30), *P* = 0.03), **Figure 2C**. There were significant differences between treatment duration groups: 0–24 months, >24–60 months, >60–108 months, and >108 months (4.60 mg/dl (IQR: 4.45–6.28) vs. 5.25 mg/dl (IQR: 4.58–6.23) vs. 5.95 mg/dl (IQR: 5.40–7.58) vs. 7.60 mg/dl (mg/dl 5.70–8.40), *P* < 0.00001), **Figure 2D**. The levels of serum uric acid were not significantly different between males and females in the risperidone group (5.70 mg/dl (IQR: 4.95–7.25) vs. 6.10 mg/dl (IQR: 4.48–7.23), *P* = 0.95), **Figure 2B**. The fasting uric acid concentrations were not associated with the gender. There were no differences of serum uric acid levels between the exclusive risperidone group and the co-medication groups (5.70 mg/dl (IQR: 4.60–7.05) vs. 5.80 mg/dl (IQR: 5.20–7.30), *P* = 0.89), **Figure 2E**.

**Table 2** shows the correlation between uric acid level and continuous variables of anthropometric, clinical, and biomedical parameters. Serum uric acid levels were positively correlated with age (*r*_s_ = 0.53, *P* < 0.0001) and risperidone dose (*r*_s_ = 0.23, *P* = 0.01) and duration of treatment (*r*_s_ = 0.43, *P* < 0.0001). Also, our study found significant positive correlations between serum uric acid level and BMI (*r*_s_ = 0.62, *P* < 0.0001), waist circumference (WC) (*r*_s_ = 0.27, *P* = 0.003), triglyceride (TG) levels (*r*_s_ = 0.36, *P* < 0.0001), TG/HDL-C (*r*_s_ = 0.47, *P* < 0.0001), insulin levels (*r*_s_ = 0.19, *P* = 0.04), HOMA-IR (*r*_s_ = 0.20, *P* = 0.03), leptin levels (*r*_s_ = 0.50, *P* < 0.0001), and hs-CRP levels (*r*_s_ = 0.37, *P* < 0.0001). There were significant negative correlations between serum uric acid levels and HDL cholesterol levels (*r*_s_ = –0.37, *P* < 0.0001) and adiponectin levels (*r*_s_ = –0.67, *P* < 0.0001). Serum uric acid levels did not correlate with LDL cholesterol levels (*r*_s_ = –0.18, *P* = 0.05), and glucose levels (*r*_s_ = –0.03, *P* = 0.75), **Table 2**. Multiple linear regression analysis indicated that age (*P* = 0.003), BMI (*P* = 0.021), TG/HDL-C (*P* = 0.030) and adiponectin levels (*P* = 0.001) were significantly correlated with serum uric acid levels, **Table 3**.

**Table 2:** Correlations between uric acid levels and continuous variables of anthropometric, clinical, and biomedical parameters in risperidone-treated patients with ASD (*n* = 127).

*Variables*	*Variables; Median(IQR)*	*Uric acid levels (mg/dl)*	
		
		*Correlation coefficient(r_s_) (n = 127)*	*P-value*
Age (years)	10.20 (8.60–13.80)	0.53	<0.0001^∗^
Risperidone dose (mg/day)	1.00 (0.50–1.50)	0.23	0.01^∗^
Treatment duration (months)	61.27 (39.83–87.97)	0.43	<0.0001^∗^
BMI	19.20 (15.71–24.86)	0.62	<0.0001^∗^
Waist circumference (cm)	70.00 (56.50–84.00)	0.27	0.003^∗^
LDL cholesterol (mg/dl)	106.00 (86.00–125.00)	-0.18	0.05
Triglyceride (mg/dl)	76.00 (59.00–111.00)	0.36	<0.0001^∗^
HDL cholesterol (mg/dl)	51.00 (43.00–64.00)	-0.37	<0.0001^∗^
TG/HDL-C	1.48 (1.02–2.28)	0.47	<0.0001^∗^
Glucose (mg/dl)	85.00 (82.00–91.00)	-0.03	0.75
Insulin (μIU/ml)	5.59 (2.31–10.28)	0.19	0.04^∗^
HOMA-IR	1.24 (0.49–2.16)	0.20	0.03^∗^
hs-CRP (mg/l)	0.70 (0.17–2.30)	0.37	<0.0001^∗^
Leptin (ng/ml)	4.17 (1.58–12.18)	0.50	<0.0001^∗^
Adiponectin (ng/ml)	31.00 (18.00–44.85)	-0.67	<0.0001^∗^


*Statistical significance was calculated by Spearman’s correlation test.*

*^∗^*P*-value < 0.05.*

**Table 3:** Multiple linear regression analysis of serum uric acid levels in risperidone-treated patients with ASD (*n* = 127).

*Variables*	*Beta*	*SE*	*P-value*
Age (years)	0.264	0.035	0.003^∗^
BMI	0.238	0.028	0.021^∗^
TG/HDL-C	0.179	0.131	0.030^∗^
Adiponectin (ng/ml)	-0.302	0.008	0.001^∗^


*Variables entered on method: age, risperidone dose, treatment duration, BMI, WC, LDL-C, TG, HDL-C, TG/HDL, glucose, insulin, HOMA-IR, hs-CRP, leptin, and adiponectin.*

**R*^2^ = 0.539, Adjusted *R*^2^ = 0.518, *P* < 0.0001*

*Statistical significance was calculated by Stepwise method.*

*^∗^*P*-value < 0.05.*

## Discussion

The present study is the first study on the prevalence of hyperuricemia in ASDs patients, and it is the first study on the impact of serum uric acid on metabolic adverse effect in ASDs children and adolescents treated with risperidone. The increased uric acid concentrations were significantly associated with adolescent patients treated with risperidone, which is concordance with the previous study in the pediatric population (Cardoso et al., 2013). The levels of uric acid were significantly higher in the risperidone treatment group than in the risperidone-naïve group (5.70 mg/dl vs. 5.35 mg/dl, *P* = 0.01). However, the levels of uric acid in the risperidone-naïve group were higher than in the normal children and adolescent group (Cardoso et al., 2013) (5.35 mg/dl vs. 4.19 mg/dl). The frequencies of hyperuricemia (>5.5 mg/dl) were differences among the risperidone group, the risperidone-naïve group, and the normal children and adolescent group (Cardoso et al., 2013) (57.50% vs. 44.70% vs. 12.40%). Many studies demonstrated that treatment with olanzapine was associated with the increase in uric acid concentrations (Tohen et al., 2007, 2008; Kryzhanovskaya et al., 2009). In this study, it was found that the treatment with risperidone was associated with increased levels of uric acid. A higher dose of risperidone and/or a longer treatment time are associated with higher levels of serum uric acid. However, the previous studies reported that Mitochondrial and purinergic dysfunction have been associated with ASD (Frye, 2015; Lindberg et al., 2015). As shown by Chauhan et al. (2011), the frontal lobe, cerebellum, and temporal lobe in post-mortem brains of children with autism had diminished expression of mitochondrial electron transport chain proteins. Page and Coleman found the *de novo* purine synthesis is increased 3–4-fold in the hyperuricosuric autistic patients compared to normal controls (Page and Coleman, 2000). Therefore, the altered uric acid concentrations in patients with ASD may be the result of the disease itself. The treatment with antipsychotics may be directly or secondarily the result of the adverse treatment-induced hyperuricemia.

Hyperuricemia is associated with the increased risk of cardiovascular disease. Increased blood uric acid concentrations are correlated with obesity (Godin et al., 2015), inflammation (Baldwin et al., 2011), type 2 diabetes (Dehghan et al., 2008), dyslipidemia (Peng et al., 2015; Stelmach et al., 2015), and the metabolic syndrome (Sagodi et al., 2015). In this study, correlations with obesity were found. BMI was positively correlated with serum uric acid levels, consistent with the results in Chinese (Zhang et al., 2014) and Taiwanese (Lu et al., 2008) pediatric populations. Ford et al. (2007) found hyperuricemia related to BMI and dyslipidemia in US children and adolescents. Stelmach et al. (2015) reported results similar to the present study showing that increased serum uric acid levels were correlated with increased serum TG levels and increased TG/HDL-C ratios and decreased HDL-C levels. Multiple regression analysis confirmed the significant positive correlation between serum uric acid and TG/HDL ratio. There is an explanation for the relationship between uric acid and TG in that the fatty acids synthesis in the liver is associated with the *de novo* synthesis of purines, consequently accelerating the production of uric acid (Matsuura et al., 1998). Previous reports indicated that the increased TG/HDL ratio is one of the predictors of cardiovascular disease (Marotta et al., 2010; Giannini et al., 2011).

Insulin resistance seems to associate with hyperuricemia. The present study found that insulin level and HOMA-IR correlated positively with serum uric acid level, which is consistent with previous studies (Gill et al., 2013; de Miranda et al., 2015). Insulin resistance probably contributes hyperuricemia through either lower renal excretion, higher synthesis or both (de Oliveira and Burini, 2012). Thus, people with normal glycemia may have excessive uric acid levels if they are insulin resistant. Excessive production of uric acid may result in impaired endothelial function (Choi et al., 2014). In this study, it was shown that increased levels of uric acid were correlated with the elevation of hs-CRP, which is consistent with a previous study (Wasilewska et al., 2012). The hs-CRP is an essential indicator of atherosclerosis (Kamath et al., 2015) and the findings lead to the proposal that serum uric acid might be a relevant marker for cardiovascular disease.

The action of adipokines is related to uric acid concentrations as well. Increased leptin levels were associated with elevated uric acid levels (de Oliveira and Burini, 2012), and leptin might be a useful marker of the metabolic syndrome in adolescents (Gonzaga et al., 2014). The present study found that serum uric acid levels linearly increased with increased serum leptin levels, which is consistent with the previous report (Fruehwald-Schultes et al., 1999). It was suggested that uric acid may serve as a biomarker of metabolic syndrome. The earlier study showed that uric acid amplifies oxidative stress in adipocytes by decreasing adiponectin (Baldwin et al., 2011). This pro-oxidative action may accelerate adipose formation (Lee et al., 2009; Johnson et al., 2011) and contribute to insulin resistance (Furukawa et al., 2004). The study from Tamba et al. (2008) reported that increased serum uric acid levels correlated with decreased serum adiponectin levels, which is consistent with this study. Multiple regression analysis confirmed the significant inverse correlation between serum uric acid levels and adiponectin levels. Decreased plasma adiponectin levels are closely related to obesity-related diseases such as dyslipidemia, type 2 DM and cardiovascular disease.

Limitation of this study includes that the data analysis was restricted due to the cross-sectional design of the study. To confirm the risperidone effect on serum uric acid levels in the patients with ASD, a longitudinal prospective study is necessary.

## Conclusion

In summary, the increased uric acid concentrations were associated with adolescent patients treated with risperidone. Fasting uric acid levels were significantly higher in ASD patients treated with risperidone than in the risperidone-naïve patients. The higher dose of risperidone and/or the longer treatment time were associated with the increased uric acid levels. The treatment with antipsychotics may be directly or secondarily the result of the adverse treatment-induced hyperuricemia in ASD patients. Our findings indicate that hyperuricemia is associated with metabolic adverse effects. Clinicians should be aware of the possible consequences of hyperuricemia in children and adolescents with autism spectrum disorder treated with risperidone.

## Ethics Statement

Ramathibodi Ethics Committee, Bangkok, Thailand (MURA2011/541) After an overnight fast, blood samples were obtained before the morning dose. Serum from clotted whole blood used for measurement of serum uric acid and metabolic parameters.

## Author Contributions

CS, NV, PL, NaN, and WK designed the research study. WK diagnosed and recruited the risperidone-naïve patients with ASD. PL and NaN diagnosed and recruited the patients with ASD treated with risperidone. NaN, WK, NoN, AP, BC, and YH collected the clinical data, performed laboratory part and evaluated the results. NV, NoN, and PS analyzed the data. NV and CS wrote the manuscript. CS, PL, and PS contributed to the discussion and reviewed/edited manuscript. All authors performed the agreement to be accountable for all aspects of the work. All authors contributed to the final approval of the version to be published.

## Conflict of Interest Statement

The authors declare that the research was conducted in the absence of any commercial or financial relationships that could be construed as a potential conflict of interest.
